# Child – parent agreement on reports of disease, injury and pain

**DOI:** 10.1186/1471-2458-6-276

**Published:** 2006-11-08

**Authors:** Gunilla M Brun Sundblad, Tönu Saartok, Lars-Magnus T Engström

**Affiliations:** 1 Dept of Molecular Medicine and Surgery, Section of Orthopaedics and Sports Medicine, Karolinska Institutet, Stockholm, Sweden; 2 Dept of Orthopaedics, Visby Hospital, Visby, Sweden; 3 Stockholm Institute of Education, Stockholm, Sweden; 4 In collaboration with The Swedish School of Sport and Health Sciences Stockholm, Sweden

## Abstract

**Background:**

Studies on school students are indicating that somatic complaints and pain have increased during the past decades. Throughout this period there has been a change in methodology from proxy reports by parents to having the students themselves act as the respondents, possible explaining some of the increase in prevalence. The aim of this study was to compare the agreement of answers from students with answers given by their parents regarding the students' medical background and subjective rating of perceived health with specific focus on frequency of headache, musculoskeletal pain and tiredness.

**Methods:**

The participating students came from eleven different schools in Sweden. The schools were a sub sample of randomly selected schools originally participating in a larger multidisciplinary base study. Those 8^th ^grade students present at school on the test date became the subjects of the investigation. A total of 232 students answered, assisted by the test leader, a specially designed self-complete questionnaire at school. Their parents were, at the same time, contacted and 200 answered a similar mailed-out questionnaire. One hundred and eighty-six (186) corresponding student-same parent questionnaires were registered for which comparisons of answers could be made and analysis conducted.

**Results:**

When a child is in good health, in absence of diseases, pain and injuries, his or her assessment matches up with their parent. Children and parents also showed agreement in cases of severe injuries and frequent (daily) complaints of knee pain. Less frequent headaches, back- and musculoskeletal pain and other complaints of minor injuries and less wellbeing, such as students' tiredness, were all under-reported and under-rated by their parents.

**Conclusion:**

When assessing the perceived health and wellbeing of students, their own expressions should be the basis for the data collection and analysis rather than relying entirely on parental reports.

## Background

Somatic complaints and pain, in children and adolescents, has been under-recognized and under-treated in the past [1,2]. Recent studies are showing an increase in self-reported prevalence [2] often with lengthy duration [3,4], clustering of complaints [3,5], as well as a pain pattern of recurrence [3]. The decline in perceived health increases with age and is more evident for girls than boys [2-8]. All the studies, cited above, were based on self-reported data from school-aged children.

According to a number of authors, the so called "gold standard" for assessing health related parameters is self-reports [6,9,10]. Yet, self-reporting by means of questionnaires or interviews has in the past only scarcely been used as an investigative tool with a population of children and adolescents. Thus, evaluation of children's health and pain has most often been based on parental responses [9,11]. Whether the above mentioned increase in somatic complaints and pain is due to an actual increase or a consequence of changes in reporting methodology, from parent to child, has to the best of our knowledge not yet been confirmed. The agreement of child self-report and parent report on pain in clinical settings has been described previously [12-14]. In a systematic review conducted by Eiser and Morse in 2001 [15] no more than two out of fourteen reviewed studies addressed the subject matter in a general non-clinical population.

### Aims

The aim of this study was to compare the agreement of answers from students, divided by gender, with answers given by their parents regarding medical background, which included questions addressing sustained injuries, diseases and handicaps and their subjective rating of perceived health with specific focus on frequency of headache, musculoskeletal pain and tiredness.

## Methods

### Participants

The participating students came from randomly selected (by The Swedish Bureau of Statistics), schools in Sweden, representing different geographical and socioeconomic regions. They were originally part of a wider, multidisciplinary base study conducted during the spring of 2001, where 1975 students, from grades 3, 6 and 9 participated [16,17].

The present study was carried out a year and a half later, during four weeks in October 2002, with a sub sample of the original 6^th ^grade classes from eleven schools. The students had entered 8^th ^grade and were age 14 at the onset of the year. Only those students present at school on the test days, were included in the investigation. Parents or legal guardians of the students received written information outlining the study and they gave their signed informed consent before the study commenced. The parents were also invited to participate in the study through answering similar questions as the students in a mailed-out separate questionnaire with a prepaid envelope to return.

In total 232 students completed their questionnaire and 200 parental responses were collected. The majority of the parental questionnaires were completed by the mother/stepmother (86%) and thereafter by the father/stepfather (13%). One questionnaire was completed by another female adult family member (0.5%) and there was one missing answer (0.5%). One hundred and eighty-six (84 girls: 45% and 102 boys: 55%) corresponding student-same parent questionnaires were registered for which comparisons of answers could be made and analysis conducted. This gives a corresponding same child same parent response rate of 82%.

The identity of the students and parents was coded to ensure anonymity. The base study, including permission for the follow-up study was approved by the Ethical committee at the Karolinska Institutet, Stockholm, Sweden (Ref. no. 00–416).

### The questionnaire

The principal investigator (G.B.S.) visited each selected school, administering and assisting the students while responding to a specially designed self-complete questionnaire. The questionnaire was, with minor revision, identical to the questionnaire answered by the same students in the base study (2001). A more detailed description of the questionnaire and the base study has been presented earlier [16,17]. Questions addressed their a) medical background, i.e. handicaps, chronic or prolonged diseases, and if any recent surgeries or fractures, requiring a cast, had occurred since the onset of the fall term; b) injuries and accidents during the recall period and since the base study, including information of site of, type of injury and setting; and c) perceived health. All students reporting an injury orally clarified their injury with the principle investigator so it complied with the definition and recall period. As a measure of the students' subjective well-being they were asked to recall their perceived health "since the onset of the fall term", i.e. mid August until the testing date, in October. Thus the recall period was 7–11 weeks. The students answered by grading on a five-point Likert scale [(1) never or almost never, (2) now and then, (3) often (every week), (4) very often, and (5) always] how often they suffered from headaches, abdominal-, back-, and/or musculoskeletal pains. Furthermore, they were asked if they frequently felt stressed, sad, and lonely and if they had problems sleeping or often felt tired.

The parents' questions were derived from the students' and addressed the same three parts, the students' medical background, their injuries and accidents and questions of perceived health, i.e. headaches, sense of fatigue, pain symptoms not attributed to an injury or disease in addition to back or knee-pain, as well as questions of perceived general health status.

### Reliability

Reliability is associated with the accuracy, consistency as well as the repeatability of a test e.g. questionnaire [18,19]. A reliability coefficient differentiates between the ratios of measured variance that is a true score from a random error. To test for reliability the same subject must answer the questionnaire at least once within no longer time than four weeks [18]. The health questionnaire used in this study was tested in a test-retest procedure. For ordinal variables, a comparison was made using the statistical procedure of Spearman Correlation and Intra class coefficient (ICC Alpha). The strength of agreement was good to very good (Cohen 1988 cited in [20,21]) with values above 0.8 (ICC: 0.9) for pain variables and above 0.9 (ICC: 0.9) for sleeping problems and tiredness. In spite of statistical tests, a low test-retest score may reflect actual changes in feelings or opinions, and on the other hand, a high score can be due to recollection of answers earlier given. In the test-retest study eight students reported that an injury had occurred during the recall period. One student failed to complete the questionnaire at the second occasion. The other seven students gave an identical answer on 99% of the 54 questions/items given.

### Validity

Steps to secure validity includes initial review from experts in the field, pilot testing with subjects, resembling the target group, and assuring the test subjects anonymity and confidentiality [18]. For content validity the present questionnaire was constructed in collaboration with a pediatrician and orthopedic surgeons trained in sports medicine. The survey, at all stages coded for anonymity, was first pre-tested for relevance and comprehension by school students of the corresponding age groups, and thereafter in a pilot study in November 2000 with 103 students from grade 3, 6 and 9.

### Statistics

The strength of agreement between responses from student and their matching parent was studied by means of absolute agreement. Absolute agreement is the shared positive and negative answers from both students and parents divided by total number of responses presented in percent. Agreement was also analyzed with the Kappa coefficient. Kappa corrects for chance and takes into account both the observed and expected value on the diagonal of a cross tabulation. For ordinal variables weighted Kappa was calculated. Weighted kappa includes weights given to values according to their distance from the diagonal so to account for the magnitude of disagreement [22]. Descriptive statistics, with frequencies of answers was used in those cases where the students' and parents' questions did not share the same format.

For all analyses, the statistical significance was set at p < 0.05.

The questionnaires were converted into a database using the SPSS (Statistical Package for the Social Sciences (SPSS 11.0, Chicago, IL, USA) computer software. For quality control, both the students' and parents' questionnaires were re-read and compared to the database to help establish the highest possible level of accuracy.

## Results

### Medical background

Twice as many students (13%, 6 girls and 18 boys) listed *handicaps or chronic diseases*, defined as hindering physical activity, compared to their parents (6.5%, parents of 7 girls and 5 boys). Students listed disorders ranging from asthma, diabetes and rheumatism to various pain conditions. Parents failed to report asthma and musculoskeletal pains noted by their child, but also heart problem, diabetes and rheumatism. Conversely, four parents listed asthma not reported by their child (See Table 1 for degree of agreement in answers).

Eight students (4 girls and 4 boys) and nine parents (parents of 3 girls and 6 boys) reported and described *a surgery or a fracture treated by a cast since the onset of the fall term *(See Table 1). However, the subsequent open ended question showed that only half of the descriptions were an agreement of the actual surgery or fracture, noted by the child and their parent.

### Injuries per specified definition and recall period (ongoing fall term)

Nearly one fifth of the students (19%, 10 girls and 26 boys) reported that they had been injured since the onset of the fall term. Almost as many parents (20%, parents of 18 girls and 20 boys) reported that their child had been injured during the same period. However, only one third of these were the exact matching child-parent recall of the injury (See Table 1).

### Injuries during the last one and a half year (since the base study) that made the student seek medical care at a hospital

Nearly one fourth (24%, 18 girls and 26 boys) of the students reported that they had sought medical care by the above definition. One fifth of the parents (21%, parents of 15 girls and 24 boys) also recalled that their child had been injured in such manner that a hospital visit was necessary. The exact child-parent agreement was only precise in half of the cases (See Table 1).

### Perceived health

*Tiredness *was rated on a Likert scale by both students and parents. Feeling tired every week or more often (3, 4 or 5 on the scale) was expressed by 24% (25 girls and 20 boys) of the students. As frequent tiredness rated by the parents was confirmed by 14% (parents of 14 girls and 12 boys). Less than half (45%) presented the same rating of tiredness between child and parent (See Table 2).

*Headaches*, experienced at least once a week was reported by 13% (14 girls and 10 boys) of the students. Four percent (n = 7) of the parents described similar rate of occurrence. Only one perfect agreement, in rating of those with daily complaints was given between same child and parent. All other were under-rated by the parents. Two parents noted that their child often suffered from headaches not reported by the child (See Table 2).

### General complaint by the child of musculoskeletal pain not attributed to an injury or illness

Perceived pain per this definition was rated by the students on the 5 point Likert scale as earlier described. Experiencing pain weekly or more often (3, 4 and 5 on the Likert scale) was expressed by 8%. The answers given by the parents were a) no (n = 125), b) now and then (n = 52), c) yes (pain) (n = 8), d) do not know (none). One parent failed to complete this question. Because the answers given were not identical due to the wording of the question, statistical analysis has not been executed. However, no agreement at all was found for girls reporting weekly to daily pain and for boys it was an agreement in two cases. The results are illustrated in Table 3.

*Back pain *was in the students' questionnaire enquired through both point prevalence and any pain since the onset of the fall term. The parental perception of whether their child complained of back pain or not was graded on a Likert scale. Point prevalence of back pain was reported by 24% (20 girls) and 18% (18 boys). Back pain during the recall period was experienced by almost as may girls (26%) (n = 22) and a few less boys (11%) (n = 11). Five parents (no gender difference) reported that their child had weekly to daily complaints of back pain. All but one was agreed by the child.

*Knee pain *was reported by the students and parents in the same manner as above. One out of every fifth student (19%, 16 girls and 22%, 22 boys) reported that they had knee pain or had had knee pain during the recall period. One third of the students (10 girls and 7 boys) had had pain for over a month. Weekly to daily knee pain was reported by parents of 6 girls and 3 boys. Total agreement was found between child and parent when complaints were "very often" but not so when lesser, more infrequent pain had been incurred.

### The perceived general health status of their child

Most parents (69%) felt that their child was in "very good" health and 31% in "good". The alternative option entitled "bad" was not comparative. No gender difference in parental answers was found in this global question.

## Discussion

In absence of pain and distress the agreement of answers between children and their parents was high, a finding shared by Waters et al. examining over 2000 adolescent-parent data sets [23]. However, when in pain, our results and those of others [11,14-16,23] have shown that parents under-report conditions and under-rate subjective complaints from their children, especially when estimating their emotional state [15,23] and recent symptoms of malaise [15].

The definition of prolonged disease and handicap varies in studies depending on motives or methods applied. Discrepancies between child and parent responses of diseases, handicaps and injuries may be due to lack of communication, the child's feelings of awkwardness about their own physical state, social desirability,"playing tough", etc. [15,25]. In addition, variations may be due to its effect on daily life, which may not be perceived as significant for child and parent Defining asthma as a handicap or prolonged disease varied. In the base study the students were asked if their asthma hindered them from being physically active at school or in their free time. Forty-eight percent of the girls and 26% of the boys found that it hindered them at times. Their perception and interpretation, along with the severity of their asthma may have influenced answers given, together with any lack of agreement with their parents on this question.

Severe injuries and conditions with obvious and stable symptoms are more likely to be rated or reported similarly by both child and parent [15,23]. In our study less than half of the descriptions were a child -parent agreement as regards to factors in their medical background and rating of tiredness. Again, this could be explained by a lack of communication as well as different recollection. Previous studies have shown that few children communicate their pain to their parents or seek medical attention for their ailments [26,27]. With reference to our earlier report on injuries during physical activity in school-children [20] some students, especially highly physically active boys, claimed that certain injuries, i.e. thigh contusions in soccer, burn-wounds from artificial turf in rugby, etc. were "part of the game", and thus expected to happen. Their ailment was not looked upon as an injury. Highly physically active students reported strains and sprains that had occurred during sport club training and competition that were often not reported by their parents. These parents rated their children's' health as excellent on most items, and unless the injury had been very serious it was not commented upon.

The impact of gender, i.e. combination of mothers' report of daughters or son and vice versa for fathers has been suggested as possibly influencing agreement [28]. General health was rated equal between genders by the parents and for the most part by the students in the present study. On the other hand, in the base study, girls rated inferior perceived health to the boys (unpublished observations), which is in line with several other recent studies [6,8,11,23]. The proportion of mothers and fathers as reporters were disproportionate in our study, which preclude further analyzes as well as drawing any firm conclusions. Nevertheless, our preliminary analyses revealed higher agreement for fathers with their children.

Limitations of the survey method applied in this study stem from that memory biases and cognitive distortions are inherent in most self-report measures and retrospective techniques [29]. A measure to enhance the quality is when it can be combined with personal instructions and one-to-one help. This may clear possible confusion, misinterpretation and eliminate social desirability and tendencies for indecisiveness or extreme responsesin answers [30]. Our students' reports of injuries were, as earlier described, complemented by a discussion with the test leader (G.B.S.). The parental responses, and their adhered to time references, were due to methodological differences obviously less controlled for.

Studies on memory and recall bias on children are few [25]. Examining the subject, McGrath et al. [6] and Haugland & Wold [31] found that children and adolescents had a good recollection and were able to accurately understand, evaluate and report their pain and complaints. Satisfying test-retest reliability (ICC or kappa value) on symptom checklists for adolescents was also established by Haugland & Wold [31]. Even children as young as age 4–5 years have proven to reliably report pain severity on various scales [14].

Regardless of age, incidents are forgotten, exaggerated and/or diminished depending on factors such as time, individual differences and past experiences [25,29].

A disadvantage with the mailed out parental questionnaires was the lack of guarantee as to who actually did answer. When analyzing collected data, as in the present study, it is important to match same child same parent since merely report frequency in answers between the two groups may possibly disclose false agreements. In spite of the acknowledged limits of self-report surveys, they provide comprehensive information in large scale research studies, saving both time and money.

Depending on circumstances and purpose of assessment both child and parent reports have shown to be valid and reliable even though children report significantly more physical complaints than their parents [19,28]. Moreover, agreement has been found to be less between healthy children and their parents compared to chronically sick children [15]. Parents own health can also influence assessment and subsequently the agreement. Waters et al. [32] found that mothers, who reported poor own health, also reported poor child health [32]. Consequently parent reports cannot be substituted for the child's, but can be valuable as a complement for a comprehensive assessment.

## Conclusion

The conclusion of this child-parent agreement study is that when a child is in good health, in absence of disease, pain and injury, his or her assessment matches up with the parent. Children and parents also showed agreement in cases of severe injuries and very frequent (daily) complaints of knee pain. Less frequent musculoskeletal pain, back pain and reports of minor injuries were all under-reported and under-rated by their parents. Children's assessment of headaches and tiredness were poorly agreed by their parents. Since both under and over treatment in clinical practices are dependent on information and assessment given by the child and/or parent, disagreement and reasons thereof are important factors to consider. This suggest that when assessing the perceived health and well-being of students, their expressions should be the basis for the data collected and analyzed rather than relying entirely on parental reports.

## Competing interests

The author(s) declare that they have no competing interests.

## Authors' contributions

GBS planned, designed, and carried out the study. Analyzed data and wrote the paper.

TS carefully and critically read and discussed the paper. L-ME initiated and planned the project. Carefully and critically read and discussed the paper as well as secured the financial background to the project. All authors read and approved the final manuscript.

## Pre-publication history

The pre-publication history for this paper can be accessed here:



## Figures and Tables

**Table 1:** Child and parent agreement regarding medical background.

	**Absolute Agreement**		*Child: *Yes *Parent: *Yes*		**Child: *Yes *Parent: *No***		**Child: *No *Parent: *Yes***		**Child: *No *Parent: *No*		**Kappa**	
	Girls	Boys	Girls	Boys	Girls	Boys	Girls	Boys	Girls	Boys	Girls	Boys
	**n**	**n**	**n**	**n**	**n**	**n**	**n**	**n**	**n**	**n**	**n**	**n**
	*%*	*%*	*%*	*%*	*%*	*%*	*%*	*%*	*%*	*%*	*%*	*%*

Handicap & diseases	**77/84**	**89/102**	**3**	**5**	**3**	**13**	**4**	**0**	**74**	**84**	0.417	0.388
	*92*	*87*	*3.5*	*5*	*3.5*	*13*	*5*	*0*	*88*	*82*		
Surgery & fractures	**80/83**	**94/100**	**2**	**2**	**2**	**2**	**1**	**4**	**78**	**92**	0.553	0.37
	*96*	*94*	*2.5*	*2*	*2.5*	*2*	*1*	*4*	*94*	*92*		
Injuries (recall period)	**64/84**	**76/102**	**4**	**10**	**6**	**16**	**14**	**10**	**60**	**66**	0.157	0.274
	*76*	*75*	*5*	*10*	*7*	*15*	*17*	*10*	*71*	*65*		
Injuries (base study, 2001)	**68/84**	**80/102**	**9**	**14**	**9**	**12**	**6**	**10**	**60**	**66**	0.406	0.417
	*82*	*78*	*10.5*	*13*	*10.5*	*12*	*7*	*10*	*72*	*65*		

Absolute agreement = Cell 1* + 4** divided by number of subjects.

Number of subjects in bold and percent in italic.

**Table 2:** Tiredness and Headache

	**Absolute agreement**		**Never/almost never (1)**		**Now and then (2)**		**Often (weekly) (3)**		**Very often (4)**		**Always (5)**		**Weighted kappa**	
	**Girls**	**Boys**	**Girls**	**Boys**	**Girls**	**Boys**	**Girls**	**Boys**	**Girls**	**Boys**	**Girls**	**Boys**	**Girls**	**Boys**
			**n**	**n**	**n**	**n**	**n**	**n**	**n**	**n**	**n**	**n**		

**Tiredness**	39% 33/82	50% 49/99	3	21	24	25	5	2	0	1	0	0	0.29	0.4
**Headache**	51% 42/82	63% 62/99	18	39	24	22	0	1	0	0	0	0	0.37	0.42

Student and parent agree that state is present in Likert scale rating from 1–5:

**Table 3:** Musculoskeletal pain

	**Never, almost never **(1)		**Now and then **(2)		**Daily complaints to once a week **(3,4 & 5)	
	
	Girls	Boys	Girls	Boys	Girls	Boys
**Students rating**	**48**	**62**	**30**	**31**	**6**	**9**
	*57*	*61*	*36*	*30*	*5*	*4*
	**No**		**Now and then**		**Yes**	
**Parents rating**	**58**	**67**	**24**	**28**	**2**	**6**
	*69*	*63*	*29*	*28*	*2*	*6*

General complaint by the child of musculoskeletal pain not attributed to an injury or illness rated on a 5 point Likert scale by the students and by the parents by the alternatives; no, now and then and yes. Number of subjects in bold and percent in italic.
